# Day hospital Mentalization-based treatment versus intensive outpatient Mentalization-based treatment for patients with severe borderline personality disorder: protocol of a multicentre randomized clinical trial

**DOI:** 10.1186/s12888-014-0301-0

**Published:** 2014-11-18

**Authors:** Elisabeth MP Laurenssen, Maaike L Smits, Dawn L Bales, Dine J Feenstra, Hester V Eeren, Marc J Noom, Maartje A Köster, Zwaan Lucas, Reinier Timman, Jack JM Dekker, Patrick Luyten, Jan JV Busschbach, Roel Verheul

**Affiliations:** Viersprong Institute for Studies on Personality Disorders (VISPD), Halsteren, the Netherlands; Arkin, Amsterdam, the Netherlands; Department of Psychiatry, section Medical Psychology and Psychotherapy, Erasmus MC, Rotterdam, the Netherlands; Department of Psychiatry, Zaanstad Medical Centre (ZMC), Amsterdam, the Netherlands; NPI specialist in personality problems, Amsterdam, the Netherlands; Lentis, Groningen, the Netherlands; Department of Clinical Psychology, VU University Amsterdam, Amsterdam, the Netherlands; Faculty of Psychology and Educational Sciences, University of Leuven, Leuven, Belgium; Research Department of Clinical, Educational and Health Psychology, University College London, London, UK; De Viersprong, Halsteren, the Netherlands

**Keywords:** Mentalization-Based Treatment, Borderline personality disorder, Cost-effectiveness, Randomized clinical trial, Treatment dosage

## Abstract

**Background:**

Borderline personality disorder (BPD) is associated with a high socioeconomic burden. Although a number of evidence-based treatments for BPD are currently available, they are not widely disseminated; furthermore, there is a need for more research concerning their efficacy and cost-effectiveness. Such knowledge promises to lead to more efficient use of resources, which will facilitate the effective dissemination of these costly treatments. This study focuses on the efficacy and cost-effectiveness of Mentalization-Based Treatment (MBT), a manualized treatment for patients with BPD. Studies to date have either investigated MBT in a day hospitalization setting (MBT-DH) or MBT offered in an intensive outpatient setting (MBT-IOP). No trial has compared the efficacy and cost-effectiveness of these MBT programmes. As both interventions differ considerably in terms of intensity of treatment, and thus potentially in terms of efficacy and cost-effectiveness, there is a need for comparative trials. This study therefore sets out to investigate the efficacy and cost-effectiveness of MBT-DH versus MBT-IOP in patients with BPD. A secondary aim is to investigate the association between baseline measures and outcome, which might improve treatment selection and thus optimize efficacy and cost-effectiveness.

**Methods/Design:**

A multicentre randomized controlled trial comparing MBT-DH versus MBT-IOP in severe BPD patients. Patients are screened for BPD using the Structured Clinical Interview for DSM-IV Axis II Personality Disorders, and are assessed before randomization, at the start of treatment and 6, 12, 18, 24, 30 and 36 months after the start of treatment. Patients who refuse to participate will be offered care as usual in the same treatment centre. The primary outcome measure is symptom severity as measured by the Brief Symptom Inventory. Secondary outcome measures include parasuicidal behaviour, depression, substance use, social, interpersonal, and personality functioning, attachment, mentalizing capacities, and quality of life. All analyses will be conducted based on the intention-to-treat principle. Cost-effectiveness will be calculated based on costs per quality-adjusted life-year.

**Discussion:**

This multisite randomized trial will provide data to refine criteria for treatment selection for severe BPD patients and promises to optimize (cost-)effectiveness of the treatment of BPD patients.

**Trial registration:**

NTR2292. Registered 16 April 2010.

## Background

Borderline personality disorder (BPD) is among the most prevalent mental disorders [1,2]. The inability to mentalize, particularly in emotional interactions, is considered to be one of the key problems in BPD [3]. Mentalizing refers to “the mental process by which an individual implicitly and explicitly interprets the actions of himself and others as meaningful on the basis of intentional mental states such as personal desires, needs, feelings, beliefs, and reasons” ([3], p21). Patients with BPD typically suffer from severe impairments in this capacity, resulting in emotional instability, impulsive behaviour, and vulnerability in interpersonal and social interactions [3]. Severe BPD patients almost invariably show high psychiatric comorbidity, particularly depression, anxiety or eating disorders, substance abuse [4-6], various other personality disorders [4,7-12] and high levels of acting-out (e.g., suicidality) [13]. BPD is also associated with seriously impaired quality of life [1] and a high economic burden of disease [14]. Lifetime risk for completed suicide associated with BPD is up to 10% [13]. Together, these findings emphasize the need for the development and dissemination of effective treatments for this severe disorder.

Psychotherapy is considered to be the treatment of choice for BPD patients [1,2]. Several guidelines [2,15-19] recommend outpatient psychotherapy such as Transference Focused Psychotherapy [20,21], Dialectical Behaviour Therapy [22], Schema-Focused Therapy [23], and Mentalization-Based Treatment (MBT) [3] for BPD.

This study focuses on MBT. MBT, which was developed by Bateman and Fonagy [3,24,25] in the United Kingdom, is a promising evidence-based treatment that has its roots in attachment theory. The aim of MBT is to help patients develop an increasingly robust mentalizing process within everyday interpersonal interactions. A day hospital MBT (MBT-DH) [26-28] and an intensive outpatient MBT (MBT-IOP) [29] programme have been developed and empirically evaluated. Although the programmes are similar in length (18 months, consisting of a 12 month treatment and 6 months maintenance phase), they differ markedly in intensity, particularly with regard to the role of group therapy. The treatment phase of MBT-DH consists of a five days per week day hospital treatment that comprises daily group psychotherapy, weekly individual psychotherapy, individual crisis management from a mentalizing perspective, art therapy twice a week, mentalizing cognitive therapy, and writing therapy. MBT-IOP consists of group psychotherapy once a week, weekly individual psychotherapy, and individual crisis management from a mentalizing perspective.

Both treatment programmes have been empirically evaluated in randomized controlled trials (RCTs), but none of these trials has directly compared MBT-IOP and MBT-DH. In a first trial [26-28], 38 BPD patients were randomized to either MBT-DH or treatment as usual (TAU). TAU consisted of standard treatment offered in the UK in general psychiatric services and consisted of (a) regular psychiatric review with a senior psychiatrist when necessary (on average twice a month), (b) inpatient admission when necessary, with discharge to nonpsychoanalytic psychiatric partial hospitalization focusing on problem solving, followed by (c) outpatient and community follow-up as standard aftercare [26]. Results after 18 months showed that MBT-DH was superior to TAU on all major outcome variables; that is, depressive symptoms, suicide attempts and self-harm, number of inpatient days, and social and interpersonal functioning. These results were maintained during an additional 18-month follow-up period [30]. At 5-year follow-up after discharge, the MBT-DH group showed further improvements on suicidality, diagnostic status, service use, use of medication, global functioning scores above 60 (Global Assessment of Functional Scale; GAF) and vocational status [28]. For example, whereas 74% of the patients in the TAU condition made at least one suicide attempt, only 23% in the MBT-DH group did so. At the end of the follow-up period, 13% of the MBT-DH patients met diagnostic criteria for BPD, compared with 87% of the TAU group. This trial also yielded data concerning health costs. Before treatment, the total health-related costs for the MBT-DH group ($44,947) and the TAU group ($52,563) were comparable; after 18 months of treatment, costs were reduced to $27,303 in MBT-DH and $30,976 in TAU. During the 18-month follow-up, costs further diminished sharply in the MBT group. At 18-month follow-up, the total health-related costs in the MBT-DH group were one-fifth of costs for patients in the TAU condition ($3,183 for MBT-DH versus $15,490 for TAU) [30]. However, this study did not include a formal cost-effectiveness analysis.

Two other studies provide further support for the efficacy of MBT-DH. An RCT in Denmark investigated the efficacy of MBT-DH compared with a less intensive manualized supportive group therapy combined with psychoeducation and medication treatment in patients diagnosed with BPD [31]. In total, 58 patients were randomly allocated to MBT-DH and 27 patients to the specialist combined treatment. Results showed that both MBT-DH and the less intensive supportive group therapy led to significant improvements on a variety of psychological and interpersonal measures, e.g., general functioning, depression, social functioning and number of diagnostic criteria for BPD [32], with moderate to large effect sizes (*d* = 0.5 to 2.1). MBT-DH was superior only on therapist-rated GAF [31] after two years of treatment. No follow-up or cost-effectiveness data are yet available from this trial. A naturalistic effectiveness study in the Netherlands [33] investigated the effectiveness of 18-month, manualized MBT-DH in 45 patients with severe BPD and high comorbidity in terms of Axis I and Axis II disorders. Results showed significant improvements in symptomatic distress, social and interpersonal functioning, and personality pathology and functioning; with moderate to large effect sizes (*d* = 0.7 to 1.7). In addition, the Netherlands study showed that care consumption reduced significantly during and after treatment. However, the lack of a control group limits the ability to draw conclusions about the effectiveness of MBT-DH.

Finally, with regard to MBT-IOP, an RCT [29] showed that this less intensive treatment programme was more effective than structured clinical management in an RCT with 134 BPD patients. Substantial improvements were observed in both treatment conditions across all outcome variables. However, the MBT-IOP group showed a steeper decline on both self-reported and clinically significant problems, including suicide attempts and hospitalization. Cost analyses were not included in this trial.

As noted, a ‘head to head’ cost-effectiveness trial directly comparing MBT-IOP with MBT-DH has not yet been conducted. An indirect comparison between MBT-DH and MBT-IOP based on the UK trials [26,29] is hampered by pretreatment patient differences; with the patients in the MBT-DH trial evidencing substantially higher levels of symptomatic distress, depression and interpersonal problems (between-group difference *d* = 0.7 to 0.8). This stresses the need for a comparative trial that also compares the cost-effectiveness of MBT-DH and MBT-IOP, particularly as MBT-DH may be approximately twice as expensive as MBT-IOP [34].

A further reason why a direct comparison trial is currently indicated relates to the current shift in psychotherapy research from the identification of effective treatments to the search for variables that might predict treatment response, with the hope of better matching patients to specific treatments (i.e., to identify “what works for whom”) [35]. Various patient characteristics have been proposed and studied as predictors of treatment outcome with MBT. Furthermore, changes in mentalizing capacities and attachment have been proposed as mechanisms of change in MBT. Little is known, however, about predictors of treatment and mechanisms of change in the treatment of BPD patients, and with MBT in particular. Bateman and Fonagy [36] found pretreatment severity, defined in terms of the number of personality disorders, to be a possible indicator for more favorable treatment outcomes in specialist treatment. No study to date has investigated purported mechanisms of change in MBT. The current trial therefore aims to identify potential predictors of response in both treatments with the aim to facilitate treatment selection and optimize (cost)-effectiveness. Furthermore, it aims to investigate mechanisms of change in both treatment programmes.

### Research aims and hypotheses

The primary aim of the present study is to investigate the efficacy of MBT-DH in comparison to MBT-IOP. The primary outcome measure is symptom severity as measured by the Brief Symptom Inventory [37,38]. Secondary outcome measures include parasuicidal behaviour, depression, substance use, social, interpersonal and personality functioning, attachment, mentalizing capacities, and quality of life. We expect that patients in both MBT-DH and MBT-IOP will improve, but that MBT-DH will outperform MBT-IOP because of its higher dosage.

A secondary aim is to investigate the cost-effectiveness of both treatments based on costs per quality-adjusted life-year (QALY). We expect that the greater benefits of MBT-DH will not outweigh the lower costs of MBT-IOP. Therefore, we expect MBT-IOP to be more cost-effective than MBT-DH.

Third, we aim to explore the role of pretreatment variables in predicting treatment outcome in both interventions, with the aim to optimize treatment selection and thus (cost-)effectiveness.

Finally, this study aims to investigate whether MBT is associated with changes in mentalizing capacities and attachment and whether such changes predict treatment outcome.

## Methods/design

### Design

**Figure 1:**
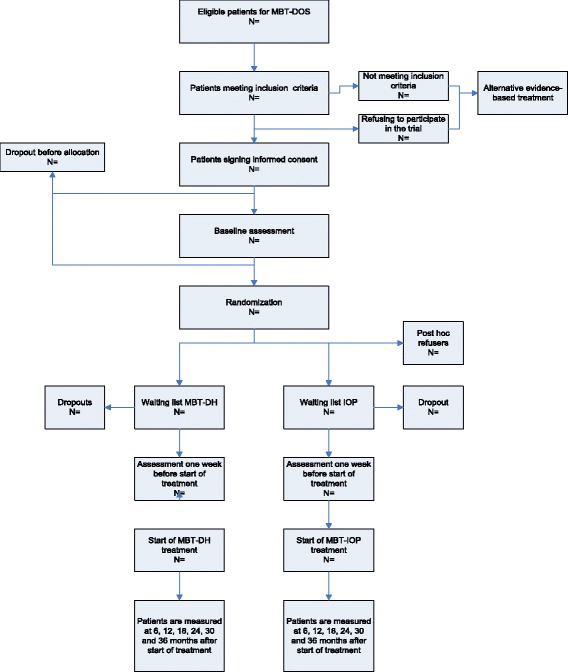
**Patient flow chart.**

This study is a multisite RCT comparing the efficacy and cost-effectiveness of MBT-DH versus MBT-IOP in the treatment of BPD (see Figure 1). Patients are randomly allocated to either MBT-DH or MBT-IOP and assessed before randomization, before start of treatment and subsequently every 6 months up to 36 months after start of treatment.

Initially, five mental health care centres specializing in the treatment of BPD agreed to participate in this study (NPI, Amsterdam; GGZinGeest, Amsterdam; De Viersprong, location Bergen op Zoom; De Viersprong, location Amsterdam; and Lentis, Groningen). NPI, GGZinGeest, and De Viersprong location Bergen op Zoom all agreed to provide two MBT-DH and two MBT-IOP groups. De Viersprong location Amsterdam and Lentis both agreed to provide one MBT-DH group and one MBT-IOP group. Before the actual start of the study, one of the participating centres (GGZinGeest) could no longer offer MBT-DH because of a reorganization of its clinical services, and accordingly withdrew from participating in the study. Due to implementation problems, NPI reduced its capacity to one MBT-IOP group and one MBT-DH group. De Viersprong location Amsterdam, however, has been able to expand its capacity to two MBT-DH groups and two MBT-IOP groups. Overall, NPI, De Viersprong (locations Bergen op Zoom and Amsterdam) and Lentis will provide a total of 6 MBT-DH and 6 MBT-IOP groups. The recruitment of patients began in January 2012 and ended in June 2014.

### Ethics

The Medical Ethical Committee of Erasmus Medical Center, Rotterdam, the Netherlands, has approved this study, registered under NL38571.078.12.

### Participants and eligibility criteria

The target group consists of adult patients (18+) with BPD, consecutively referred for treatment by general practitioners, mental health care institutions, private practices and general hospitals.

#### Inclusion criteria for MBT treatment and for this study

Inclusion criteria are (a) a BPD diagnosis as assessed using the Structured Clinical Interview for DSM-IV Axis II Personality Disorders (SCID-II) [39], (b) 18 years or older, (c) adequate mastery of the Dutch language and (d) travel time to the MBT setting less than 1 hour.

#### Exclusion criteria for this study

Exclusion criteria for MBT are kept to a minimum because the treatment aims to reach a broad population of BPD patients. In this study, only patients with (a) a diagnosis of autism spectrum disorders, chronic psychotic disorder or organic brain disorder that interferes significantly with the ability to mentalize; (b) intellectual impairment (IQ <80 as measured by the Wechsler Adult Intelligent Scale–III) [40]; or (c) a diagnosis of antisocial personality disorder in combination with a history of severe physical violence are excluded. In addition, patients who have had a stable job for a period of at least 2 years for a minimum of 15 hours a week, and/or are caregivers with main responsibility for children under 4 years of age, are allowed to participate in the randomized study at their own choice. Because of ethical reasons (i.e. randomization to MBT-DH cannot be combined with a job or being the main carer for children because of the fixed time commitment involved), these patients were not seen as refusers, and were given the option to enter MBT-IOP directly.

### Procedure

All patients referred to one of the mental health care centres are invited for a standard intake interview, which includes standardized assessment by means of the Structured Clinical Interview for DSM-IV Axis I disorders (SCID-I) [41] and SCID-II [39]. All patients eligible for MBT treatment (with a BPD diagnosis and aged ≥18 years) are given both written and verbal information about the study. Eligible patients are given 1 week to consider participation in the trial. Patients who are willing to participate in the study are asked to sign an informed consent sheet and complete the baseline assessment. Next, they are randomized to either MBT-DH or MBT-IOP by an independent researcher using a computerized 1:1 algorithm. Patients are informed about their treatment allocation and placed on a waiting list. One week prior to start of the main treatment phase, and subsequently at 6-months intervals up to 36 months after the start of treatment, patients complete an assessment battery as described below. Patients who are excluded from the trial on the basis of exclusion criteria, or who refuse to participate in the trial, are ideally assigned to an alternative evidence-based treatment. Excluded and refusing patients are asked to complete the baseline assessment only, for which they sign a separate informed consent form. At 18 and 36 months, the SCID-I and SCID-II are re-administered by MSc-level psychologists. Extensive efforts are being made to collect data at the follow-up time points by means of an outreaching approach towards patients: patients are contacted by telephone, e-mail and letters (with reminders as necessary) to motivate them to attend assessments, and home visits are made if necessary.

### Treatments

MBT was developed by Fonagy and Bateman in the United Kingdom [3,24,25,42]. The term *mentalizing* refers to the ability to attend to mental states in ourselves and in others as we attempt to understand our own actions and the actions of others on the basis of intentional mental states. The aim of MBT as a therapy is to help patients develop an increasingly robust mentalizing process within the context of interpersonal interactions. Treatment begins with MBT-I, an introductory phase with psychoeducational elements. This is followed by a combination of individual and group therapy.

MBT works through continuously helping the patient to maintain or re-establish a mentalizing process while simultaneously stimulating the attachment system by balancing the level of optimal arousal. With a main focus on current rather than past experience, patients are encouraged to explore the mental states of themselves and others, to become curious about alternative perspectives, to find out more about how they think and feel about themselves and how this influences their behaviour, and how distortions in understanding themselves and others lead to problems in interpersonal functioning. The exploration and identification of emotions within multiple contexts, particularly interpersonal contexts, promotes the development of secondary representations to primary affective experiences. This enhances affect regulation and the development of a coherent sense of self. These elements form key components of mentalizing capacity. In this manner, the work in therapy addresses BPD patients' difficulties with affect, impulse regulation and interpersonal functioning. Destructive behaviour, which from an MBT perspective is understood as an attempt to maintain a sense of stability and manage incomprehensible feelings in the absence of a mentalizing capacity, is reduced as a consequence of the development of this capacity. Another important focus lies in generalizing improvements in daily life to wider social functioning. Thus, by means of improving patients’ mentalizing capacity, several domains of functioning are addressed.

Both MBT-DH and MBT-IOP have five general treatment goals: (1) engagement in therapy; (2) reduction of psychiatric symptoms, particularly depression and anxiety; (3) reduction of self-damaging, threatening or suicidal behaviour; (4) improved social and interpersonal functioning; and (5) stimulation of appropriate use of general mental health services (including prevention of reliance on prolonged hospital stays). Goals are personalized and linked to the components of the programme. Both MBT programmes are divided into three phases: (1) pretreatment, (2) main treatment, and (3) follow-up treatment [3,24,25,42].

#### Pretreatment

Patients enter the pretreatment programme until a place becomes available in the treatment to which each patient has been randomized (MBT-DH or MBT-IOP). For both MBT-DH and MBT-IOP the pretreatment programme focuses on engaging the patients in treatment and crisis management. It consists of an introductory course to MBT (MBT-I), and biweekly individual sessions with crisis planning as focus. MBT-I is an explicit mentalizing group with a psychoeducational element, which is considered an important part of the programme. The programme consists of 12 highly structured sessions that stimulate patients to consider the overall process of mentalizing and its relation to their problems, and how these problems will be addressed in MBT [25,42].

#### Main treatment

##### MBT-DH

MBT-DH is an intensive, manualized treatment for patients with BPD. It has been described in detail elsewhere [3,33]. Briefly, MBT-DH consists of a highly structured day hospitalization programme with a maximum duration of 18 months, covering five days per week. MBT-DH consists of the following components: daily group psychotherapy, weekly sessions of individual psychotherapy, individual crisis planning on indication (average: weekly for 3 months and from then on lower in frequency), art therapy twice a week, mentalizing cognitive group therapy, and writing therapy. Each week’s programme is ended with a social hour and a community meeting. Patients in the MBT-DH programme can also consult a psychiatrist upon request and medication is prescribed following American Psychiatric Association (APA) guidelines [15].

##### MBT-IOP

The main treatment phase in MBT-IOP consists of group psychotherapy twice a week, weekly sessions of individual psychotherapy, and individual crisis management on indication (average: weekly for 3 months and from then on lower in frequency). Patients can also consult a psychiatrist upon request and medication is prescribed following APA guidelines.

#### Follow-up treatment

For patients in either treatment condition, the final phase of the programme offers individually tailored stepped-down care aiming at relapse prevention, maintaining and further enhancing the gains made in mentalizing capacity, and stimulating further rehabilitative change and reintegration into society.

### Therapists

Both the MBT-DH and MBT-IOP teams consist of therapists with broadly ranging level of experience, background, and educational level. All therapists involved in the study are certified psychologists, psychotherapists, psychiatrists, nurses or art therapists. All clinicians who are involved in the study have successfully completed a certified MBT basic course, after which they receive continuous supervision in working with the MBT model.

### Treatment adherence

One of the authors, D. L. Bales, trained by Anthony Bateman, was involved in the implementation and monitoring of MBT at the different treatment sites. Adherence to the MBT treatment model is monitored in several ways. First, adherence in daily practice is monitored by means of reflections after each group therapy session, in which therapists are continuously stimulated to reflect on their adherence to the treatment model. Specifically, therapists are asked to reflect on whether their interventions have enhanced mentalizing which interventions have not achieved this, and what alternative interventions might have been more successful. Second, within both the MBT-DH and the MBT-IOP programme, biweekly team supervision focuses on review of case material to increase therapists’ comprehension of mentalizing theory and their competency in working with the principles of MBT and the spectrum of mentalizing interventions. Third, we will rate the adherence to the model of both group and individual sessions using videotaped sessions. Based on the videos, raters trained in the use of the Adherence and Competence Scale [43] will measure therapist adherence.

### Measurements

#### Demographic variables

At baseline, participants complete questions concerning demographic variables such as their marital status, living situation, religion, level of education, current job and working situation, and questions concerning the main earner in the family (relationship to the patient, annual income, occupation and source of income).

#### Trauma

The prevalence of trauma in childhood is measured by means of a Dutch translation of the short form of the Childhood Trauma Questionnaire (CTQ) [44]. The CTQ is administered only at the start of treatment (T1). The CTQ is a retrospective, self-report questionnaire that measures five categories of childhood trauma experience, including emotional, physical and sexual abuse as well as emotional and physical neglect. Each subscale is measured in 5 items rated on a 5-point Likert scale: (1) never true, (2) rarely true, (3) sometimes true, (4) often true, and (5) very often true. Each subscale score ranges from 5 (no history of abuse or neglect) to 25 (very extreme history of abuse and neglect). Cutoff scores are defined for none (or minimal) to low, low to moderate, moderate to severe and severe to extreme exposure. Moderate to severe cutoff scores were used to classify subjects as positive for a history of childhood trauma in a specific category. Cutoff scores are 13 or higher for emotional abuse, 10 or higher for physical abuse, 8 or higher for sexual abuse, 15 or higher for emotional neglect, and 10 or higher for physical neglect. Research has shown good psychometric properties for both the original CTQ version [44,45] and the Dutch translation [46].

#### Primary outcome measure

##### Symptom severity

General psychopathological symptoms are assessed with the Dutch version of the Brief Symptom Inventory (BSI) [37,38]. The BSI is the short version of the Symptom Checklist-90. It consists of 53 items covering nine symptom dimensions (somatization, obsession-compulsion, interpersonal sensitivity, depression, anxiety, hostility, phobic anxiety, paranoid ideation and psychoticism) and yields three global indices of distress: Positive Symptom Distress Index, Positive Symptom Total, and Global Severity Index (GSI). Possible GSI scores range from 0 to 4, with higher scores indicating a higher level of psychological and emotional distress. Respondents have to rate each item (e.g., “your feelings are easily hurt”) on a five-point scale ranging from 0 (not at all) to 4 (extremely), representing the intensity of distress relating to each item during the past 7 days. The reliability of the Dutch version of the BSI is good (Cronbach’s α ranging from .71 to .88, test-retest reliability ranging from r = .71 to .89). These values are comparable to the original BSI version of Derogatis [38].

#### Secondary outcome measures

##### Parasuicidal behaviour

Suicide and self-harm are assessed with the Suicide and Self-Harm Inventory (SSHI) [3]. The SSHI is a semi-structured interview assessing (a) the frequency and severity of suicidal acts in the past 6 months, and (b) the frequency and severity of acts of self-mutilation in the past 6 months. Multiple acts of self-mutilation over a short period of time – for example, frenzied self-cutting – are counted as a single act.

##### Symptomatic distress

Symptomatic distress is assessed with the Beck Depression Inventory (BDI-I), and the Measurements in the Addictions for Triage and Evaluation (MATE).

The BDI-I is used to assess depressive symptoms [47,48]. The BDI-I is a self-report instrument that consists of 21 questions concerning depressive symptoms during the past week. Each question has a set of four possible answers, ranging in intensity from 0 to 3, for example, “I don’t feel sad” (0) to “I feel so sad or unhappy that I cannot bear it anymore” (3). The total scores for the instrument are categorized: 0–9 no depression, 10–18 mild-moderate depression, 19–29 moderate-severe depression, 30–63 severe depression. The BDI has shown good psychometric qualities for both the original [47,48] and the Dutch version [49-51].

The substance use section of MATE 2.0 [52] is used to assess substance abuse and dependency. This section, which is designed as an interview, asks about the use of psychoactive substances in the past month and during the lifetime. The interviewer in this study was an MSc-level psychologist. The interrater reliability of this instrument ranges between 0.75 and 0.92 [53].

##### Attachment and mentalizing capacities

Attachment is assessed with the Experiences in Close Relationships (ECR) questionnaire. Mentalizing capacities are measured by the Reflective Functioning Questionnaire (RFQ), the Reading the Mind in the Eyes Test (RMET), and the FaceMorph task.

A Dutch translation of the Experiences in Close Relationships questionnaire (ECR) [54,55] measures adult attachment in romantic relationships. It contains two subscales: Anxiety (sample item: “I worry about being abandoned”) and Avoidance (“I feel very uncomfortable when my partner wants to have a close connection to me”) both consisting of 18 items. Individuals are asked to rate each statement on a 7-point Likert scale ranging from 1 (disagree strongly) to 7 (agree strongly). The Dutch version of the ECR was found to be a valid measure with good internal and external validity [55].

The RFQ assesses reflective functioning (a close proxy for mentalizing capacities) with 57 items that are rated on a 7-point scale (1 = strongly disagree to 7 = strongly agree). An example item is “People's thoughts are a mystery to me." As this measure is still under development, no psychometric data are yet available.

The Reading the Mind in the Eyes Test (RMET) [56] was developed as a measure of capacities in adults. Patients are shown photographs of 36 pairs of eyes, and have to match the eyes in each picture with one of four written expressions describing different emotions. In this study, a Dutch version of the RMET is being used [57].

The FaceMorph task is an experimental computerized task assessing capacities based on external features (i.e., facial expressions) of others. Morphed faces representing five emotions (happiness, sadness, anger, surprise, and fear) are shown on a screen at six different intensities (20%, 30%, 40%, 50%, 60%, and 70%) in random order. Every emotion is shown three times for each level of intensity, resulting in a total of 90 trials. Patients are asked to name the emotion they see and indicate their level of confidence in their choice on a 6-point Likert-type scale. Morphed facial emotion expressions are based on the NimStim dataset [58]. The dependent measures are accuracy, level of confidence in correct and wrong responses, and reaction time.

##### Personality pathology

Personality functioning is assessed with the Severity Indices of Personality Problems (SIPP-118) and the Personality Assessment Inventory-Borderline (PAI-BOR).

The SIPP-118 [59] is a dimensional self-report measure assessing the severity of personality pathology. The SIPP aims to assess the core components of adaptive and maladaptive personality functioning. It consists of 118 items, and comprises 16 facets that cluster into five higher-order domains: social concordance, relational functioning, self-control, responsibility, and identity integration. The SIPP-118 asks respondents to think about the past 3 months and to answer with the extent to which they agree with statements such as “I frequently say things I regret later” or “Whenever I feel something, I can almost always name that feeling”. Items are rated on a 4-point Likert-type scale ranging from 1 (fully disagree) to 4 (fully agree). Higher scores for each facet indicate better functioning. The psychometric features of the SIPP-118 are good, with evidence for good reliability (alpha coefficients ranging from .62 to .89, with a mean estimated α score of .78), convergent validity [59], and invariance of the factor structure [59].

The Dutch version of the PAI-BOR [60,61] was used to assess borderline symptomatology. The PAI-BOR is part of the Personality Assessment Inventory [60] and consists of four subscales (each with six items), which reflect four characteristics of BPD: affective instability (AI), identity problems (IP), negative relationships (NR), and self-harm (SH). There are four response categories (0 = false, 1 = slightly true, 2 = mainly true, and 3 = very true). An example item is “Sometimes I feel very empty inside”. A total PAI-BOR raw score of 38 or more indicates the presence of significant BPD features, whereas a score of 60 or more indicates typical borderline personality functioning [60]. The internal consistency (Cronbach’s α) and 6-month test-retest correlation for both the sum score and the sub domains are good.

##### Quality of life

Quality of life is measured using the EuroQol EQ-5D-3 L [62]. This self-report questionnaire provides a simple method to capture health problems according to a five-dimensional classification: mobility, self-care, usual activities, pain/discomfort, and anxiety/depression. Each dimension is divided into three levels: no problem, moderate problems, and extreme problems. The five dimensions can be summarized into a “value”, based on the preferences of the general public. These values can be used as societal weights for the calculation of Quality Adjusted Life Years (QALYs) in health economic evaluations (see also below). To calculate these societal weights, we used a Dutch validation study [63]. Adjacent to the five dimensions, the EuroQol presents a vertical visual analogue scale, ranging from 0 (worst imaginable health) to 100 (best imaginable health), on which the respondent marks the point that they feel represents their current health. The values on this scale are seen as representing patients’ values, in contrary to the societal weight based on the five dimensions. The reliability of the EQ-5D-3 L has been investigated and found to be acceptable [64], and it has shown to be sensitive to change in patients with personality disorders [14,65].

##### Social and interpersonal functioning

A Dutch translation of the Inventory of Interpersonal Problems (IIP-64) is used to assess interpersonal problems [66,67]. The IIP-64 is a self-report measure consisting of 64 items, assessing eight dimensions of interpersonal problems: (1) domineering/controlling (sample item: “It is hard for me to take instructions from people who have authority over me”), (2) vindictive/self-centred (“It is hard for me to trust other people”), (3) cold/distant (“It is hard for me to show affection to others”), (4) socially inhibited (“It is hard for me to introduce myself to someone”), (5) non-assertive (“It is hard for me to be firm when I need to be”), (6) overly accommodating (“It is hard for me to be angry at others”), (7) self-sacrificing (“It is hard for me to be angry at someone I like”), and (8) intrusive/needy (“It is hard for me to be on my own”). Respondents are asked to consider each problem and to rate how distressing that problem has been on a scale ranging from 0 (not at all) to 4 (extremely). The IIP-64 has shown good psychometric properties for both the original and the Dutch version [66,67].

##### DSM-IV Axis I and Axis II diagnoses

The Dutch version of the SCID-I [41,68] is used to assess Axis I disorders at intake and again at 36-month follow-up. The interviewer is an MSc-level psychologist, who was trained by an expert trainer in the SCID-I and SCID-II. The SCID-I has good interrater reliability (K = .85), especially when interviewers receive training as in the present study [69]. The Dutch version of the SCID-II [39,70] is used for diagnosing Axis II personality disorders at intake and at 36-month follow-up. No interrater reliability data will be collected in this study. Previous research has shown that both the original SCID-II and the Dutch version have good interrater reliability and test–retest interrater reliability in adults [71-73].

##### Costs

The intervention costs of MBT-DH and MBT-IOP will be calculated using a mixture of top-down and bottom-up approaches. The intervention costs estimates will include personnel costs, implementation costs (e.g., hosting and coaching), and any other overhead costs associated with the treatment. Medical costs beyond the intervention costs specific to MBT-DH and MBT-IOP will be calculated using the Trimbos and Institute for Medical Technology Assessment Questionnaire on Costs Associated with Psychiatric Illness (TiC-P) [74-76]). The TiC-P will be used to measure health care utilization at baseline and after 6, 12, 18, 24, and 36 months.

The first part of the TiC-P consists of questions on (1) the number of visits to, for example, a general practitioner, psychiatrist (outside MBT-DH or MBT-IOP), medical specialist, physiotherapist or alternative health practitioner; (2) the day care/hospital lengths of stay (outside MBT-DH or MBT-IOP); and (3) the use of medication in the 4 weeks prior to filling out the questionnaire [74]. These values are multiplied by the unit prices of the corresponding health care services according to the Dutch manual for costing studies in health care [76,77]. The unit prices will be adjusted to 2014 prices using the Consumer Price Index [78]. As the mean direct costs are measured per 4 weeks, we will multiply these values by 13 to obtain estimates for the annual costs.

The Tic-P also asks the patient to report any productivity losses, that is, absence from work or reduced productivity at work. This is used to estimate the so-called “friction costs”, that is, the monetary representation of the replacement of the labour. The friction-cost method takes the employer's perspective and counts as “lost” only those hours not worked until another employee takes over the patient's work. This is a more conservative estimate than the so-called “human capital method”, which relates productivity costs to the labour costs of the patient on a one-to-one basis. The choice between friction costs and human capital is still a subject of debate among economists. In this study, we chose the more conservative friction-cost method [79,80].

### Sample size and power calculation

Sample size calculation is based on the BSI, the primary clinical outcome variable in this study. We applied the mixed model ANOVA procedure described by Aberson [81], power of 0.80 and a two-sided alpha of 0.05.

We anticipated a medium-sized difference in effect size between MBT-IOP and MBT-DH based on prior research. In their RCT comparing MBT-IOP with structured clinical management, Bateman and Fonagy reported a within-group effect size for MBT-IOP of *d* =1.31 [29]. In their RCT comparing MBT-DH with usual care, the same authors reported a within-group effect size of *d* = 2.30 for MBT-DH at follow-up [26]. Hence, using an indirect comparison, the previous studies suggest at least a medium-size difference in effect (*d* = 0.50) between both treatment programmes. Applying seven repeated measures with a linear decrease of 0.5 SD more in MBT-DH than in the MBT-IOP group and an autoregressive intercorrelation structure with *r* = 0.50 between baseline and the last follow-up, 45 cases are needed in each group.

### Statistical analyses

First, to investigate potential differences between the two groups at baseline, we will use parametric and nonparametric descriptive statistics, as appropriate. For the main analyses concentrating on primary and secondary outcomes, linear growth curve models for normally distributed outcome measurements will be used, logistic regression for binary data, and Poisson regression models for ordinal data. Results will be expressed in terms of comparison of the slopes for interval data, odds ratios for binary data, and incidence rate ratios for ordinal data. Furthermore, we will perform explorative analyses regarding predictors of differential treatment outcome. All analyses will be conducted according to intention-to-treat principles.

#### Health economic evaluation

We will estimate the difference in total costs for MBT-DH compared to MBT-IOP and the difference in clinically relevant effects of the treatments. By dividing the difference in costs by the difference in effectiveness, the incremental cost-effectiveness ratio (ICER) will be estimated. The ICER represents the extra amount of money that has to be invested to gain one extra unit of effect (or, conversely, the amount of money that will be saved if one unit of effect is lost).

In the economic evaluation, all relevant costs and effects will be taken into account. This means that we will use a so-called societal perspective, which is preferred in economic evaluations in the Netherlands [82]. The costs will include *all* costs, that is, intervention costs, direct and indirect medical costs, as well as productivity losses and costs accruing elsewhere in the health care system. From the societal perspective, clinically relevant effects are those effects that are meaningful for society. Moreover, in order to be able to compare effects between different interventions in health care, effects should be expressed in generic terms. We will therefore use QALYs and will express the cost-effectiveness of each intervention and the ratio between these cost-effectiveness estimates as cost per QALY. The use of QALYs is advised in guidelines for cost-effectiveness analysis, especially when main effects are expected in quality of life [82,83].

Our primary cost-effectiveness ratio will be estimated using empirical data only and therefore a 3-year time horizon will be used, which is equal to the trial duration. Cost data are generally highly skewed, and QALY scores and ICER values are not distributed normally in most cases. Therefore, the uncertainty intervals around the mean costs, mean effects and mean ICER values will be estimated using bootstrap simulations with at least 1000 replications [84-86]. These results will be graphically presented in a cost-effectiveness plane. Various societal willingness-to-pay values will be used to estimate net monetary benefits. These net monetary benefits are then used to derive cost-effectiveness acceptability curves.

We will also explore the long-term costs and effects of MBT-DH and MBT-IOP from a societal perspective. One way of achieving this would be to use a Markov model to explore the long-term cost-effectiveness of the intervention under study. However, in a Markov model, health stages are often predefined, discrete stages, which should reflect the biological or theoretical understanding of the condition being modelled [87,88]. Because in this trial the primary outcome measures, such as the BSI, are continuous measures, it will not be possible to create an unambiguous definition of health states to model disease progression. Using heterogeneous health states may be controversial, as it means that a wide range of patients may be clustered in one health state. In order to explore the cost-effectiveness of the interventions using a Markov model in our study, we will therefore define health states on the basis of a cut-off score on one of the outcome measures such as the BSI, in order to create discrete health stages. We will extend the cost-effectiveness model results after the duration of the trial using different assumptions based on, for example, the literature. Sensitivity analysis will be performed in order to investigate the influence of those assumptions on the model results.

## Discussion

Both MBT-DH and MBT-IOP have shown promise in the treatment of patients with BPD [2,15-19]. Yet, extant studies suggest that MBT-DH may be twice as costly as MBT-IOP. This cost difference makes MBT-IOP potentially a more cost-effective treatment than MBT-DH. On this basis, there is a need for an RCT comparing the efficacy and cost-effectiveness of MBT-DH and MBT-IOP.

The present paper outlines the study protocol for such a head-to-head comparison of MBT-DH and MBT-IOP in terms of their efficacy and cost-effectiveness in the context of a multisite study in the Netherlands. The study aims to answer a number of important questions in the field. First, this will be the first trial directly comparing the efficacy of MBT-DH and MBT-IOP. Second, this promises to be the first study that entails a state-of-the-art cost-effectiveness component for both MBT-DH and MBT-IOP. Given considerable differences in treatment intensity, and thus costs of the two treatment programmes, the results of this study promise to inform decisions not only concerning treatment referral, but also with regard to maximizing the use of health care resources in a climate of decreasing health expenditure. Another important goal of the final aim of the study, which focuses on predictors of change, is to provide information that will contribute to the optimization of treatment effects and health care resources. For instance, it may well be that both treatments have equal effectiveness and differ only in terms of cost. If this were the case, health care resources should be devoted to the less costly variant (probably MBT-IOP, due to its lower intensity). However, even if it is found that both treatments differ only in terms of their costs, there may still be a subgroup of patients that may need the more intensive intervention, MBT-DH, to derive benefit. In this scenario, MBT-DH would still be indicated as a cost-effective treatment in a subgroup of patients, and it would be crucially important to identify the features of this subgroup, to enable this intervention to be offered specifically to the patients who need this more intensive treatment format. Therefore, this study also aims to identify potential predictors of response to both MBT-IOP and MBT-DH, with the aim of facilitating treatment selection.

## Abbreviations

APA: American Psychiatric Association
BDI-I: Beck depression inventory
BPD: Borderline personality disorder
BSI: Brief symptom inventory
CTQ: Childhood trauma questionnaire
ECR: Experiences in close relationships
GAF: Global assessment of functioning
GSI: Global severity index
ICER: Incremental cost-effectiveness ratio
IIP-64: Inventory of interpersonal problems
MATE: Measurements in the addictions for triage and evaluation
MBT: Mentalization-based treatment
MBT-DH: Day hospital mentalization-based treatment
MBT-I: Introductory course to mentalization-based treatment
MBT-IOP: Intensive outpatient mentalization-based treatment
PAI-BOR: Personality assessment inventory-borderline
QALY: Quality-adjusted life-year
RCT: Randomized controlled trial
RFQ: Reflective functioning questionnaire
RMET: Reading the mind in the eyes test
SCID-I: Structured clinical interview for DSM-IV axis I personality disorders
SCID-II: Structured clinical interview for DSM-IV axis II personality disorders
SIPP-118: Severity indices of personality problems
SSHI: Suicide and self-harm inventory
TAU: Treatment as usual
TiC-P: Trimbos and Institute for Medical technology assessment questionnaire on costs associated with psychiatric illness
